# Structural dynamics of *Populus euphratica* forests in different stages in the upper reaches of the Tarim River in China

**DOI:** 10.1038/s41598-020-60139-7

**Published:** 2020-02-21

**Authors:** Ning Miao, Peipei Jiao, Wenjing Tao, Maoping Li, Zhijun Li, Bin Hu, Timothy C. Moermond

**Affiliations:** 1 Key Laboratory of Bio-Resource and Eco-Environment of Ministry of Education/College of Life Sciences, Sichuan University, Chengdu, 610065 Sichuan P. R. China; 2 Xinjiang Production & Construction Corps Key Laboratory of Protection and Utilization of Biological Resources in Tarim Basin, College of Life Sciences, Tarim University, Alar, 843300 P. R. China

**Keywords:** Forest ecology, Population dynamics, Riparian ecology

## Abstract

We selected four *Populus euphratica* Oliv. forest plots (100 m × 100 m) in the upper reaches of the Tarim River in the Xinjiang Uygur Autonomous Region of China. Each of the four forest plots was chosen to represent a different growth and death stage of *P. euphratica* forest: juvenile forest, mature forest, dying forest, and dead forest. In each plot, we measured the coordinates, DBH, height, and status of all *P. euphratica* individuals. We used (1) spatial pattern analysis to explore spatial distribution patterns and associations of live trees and dead trees, (2) a random mortality model to test whether the tree death was random or non-random, and (3) a generalized linear mixed-effect model (GLMM) to analyse factors related to tree survival (or death). In the juvenile plot, live trees were significantly aggregated at all scales (*p* < 0.05); while in the mature and dying plots, live trees were more aggregated at small scales and randomly distributed at larger scales. Live trees and dead trees showed a significantly positive association at all scales in the juvenile plot (*p* < 0.05). While in the mature and dying plots, live trees and dead trees only showed a significantly positive association at scales of 0–3 m (*p* < 0.05). There was significant density-dependent mortality in the juvenile plot; while mortality was spatially random at all scales in the mature and dying plots. The distance from the river showed significantly negative correlations with tree survival (*p* < 0.01). DBH and height had significantly positive associations with tree survival in the juvenile, mature, and dying plots (*p* < 0.05). In extreme drought, dying trees appeared to be shape-shifting into more shrub-like forms with clumps of root sprouts replacing the high canopies. The shift under extreme drought stress to more shrub-like forms of *P. euphratica* may extend their time to wait for a favourable change.

## Introduction

*Populus euphratica* Oliv., the desert poplar, is a prominent component of Tugai floodplain ecosystems along river valleys in arid and semi-arid regions with a very wide range, occurring naturally from North Africa, across the Middle East and Central Asia to western China^1,2^. The *P. euphratica* forests, which are also known as Tugai forests, are the main component of the desert riparian vegetation in Central Asian inland rivers. These *P. euphratica* forests provide an important habitat for plants and animal life and harbour the highest plant biodiversity in desert regions^3–5^, which is likely also to enhance local biodiversity of animals, such as birds and insects. Additionally, these Tugai forests provide major natural resources (timber, fuelwood, livestock fodder, etc.) and provide critical environmental benefits such as increasing watershed protection, including stabilization of riverbanks and providing windbreaks to reduce erosion of sand and soil^1,4,6^.

The expansion and decline processes of *P. euphratica* forests are closely related to hydrological events (e.g., river diversion, variation of river discharge, and groundwater depth) coupled with soil properties (e.g., moisture and salinization) in riparian areas in the arid regions^1,3,7^. The *P. euphratica* forests colonized and flourished in areas along the seasonally flooded Tarim River, which is one of the largest inland rivers in the world, in the Xinjiang Uygur Autonomous Region of China^1^. These desert poplar forests declined and disappeared in areas where clearcutting and diversions of the Tarim River for irrigation resulted in a reduction of water flow and flooding frequency and in an increase in soil salinization^1,3^. Where the poplar forests disappeared, they were often replaced by *Tamarix* shrub vegetation^1,8^.

The multifunctional Tugai riparian forest ecosystem and its biodiversity have been suffering from water shortage due to climate change and human activities^3,4,9^. From 1972 to 2000, there were many land uses that led to increases in the frequency, duration, and severity of drought stress^10,11^. In 2000, China implemented a strict Natural Forest Protection Project, and the Ministry of Water Resources of China and the Xinjiang People’s Government implemented the Comprehensive Management Project of the Tarim River Watershed, including the Lower Reaches Ecological Water Conveyance Project and the Middle Reaches Water Conveyance Project^6,12^. With those projects, the *P. euphratica* forest and its watershed along the Tarim River began to be protected^12,13^. With an increase in the water conveyance since 2000, the groundwater level has risen, and the damaged riparian vegetation downstream has shown some recovery^9,10,12,14^.

Over the past decades, researchers have studied spatial distribution patterns^10,15^, regeneration patterns^2,7,16,17^, plant species diversity^18–20^, stand structure^2,21–24^, tree growth^25–27^, and restoration^14,28^ of *P. euphratica* forests. Although responses of the Tarim Desert riparian forests to different hydrological conditions are increasingly studied, the complexities of spatial distribution patterns and structural dynamics of the *P. euphratica* forest still need to be better understood. Therefore, we focused on sites of relatively undisturbed *P. euphratica* forest and addressed the following questions: (1) is tree mortality random or non-random over different growth and death stages; (2) in the different stages of *P. euphratica* forests, how do spatial patterns of live and dead trees change through time; and (3) which attributes (density, size, etc.) are related to the survival and death of *P. euphratica* in the different stages of *P. euphratica* forests.

A useful tool to characterize spatial patterns and interactions of plant communities is point patterns analysis^29^, which allows comparative estimates of the spatial distributions of mapped plant individuals in a given study area^30^. In this study, we used spatial pattern analysis of the fine-scale spatial distributions of trees to reveal structural dynamics of four different stages of *P. euphratica* forests in different growth and death patterns at different distances from the main river channel of the Tarim River in its upper reaches in the Tarim Basin of the Xinjiang Uygur Autonomous Region, China.

## Results

### Species composition

**Figure 1:**
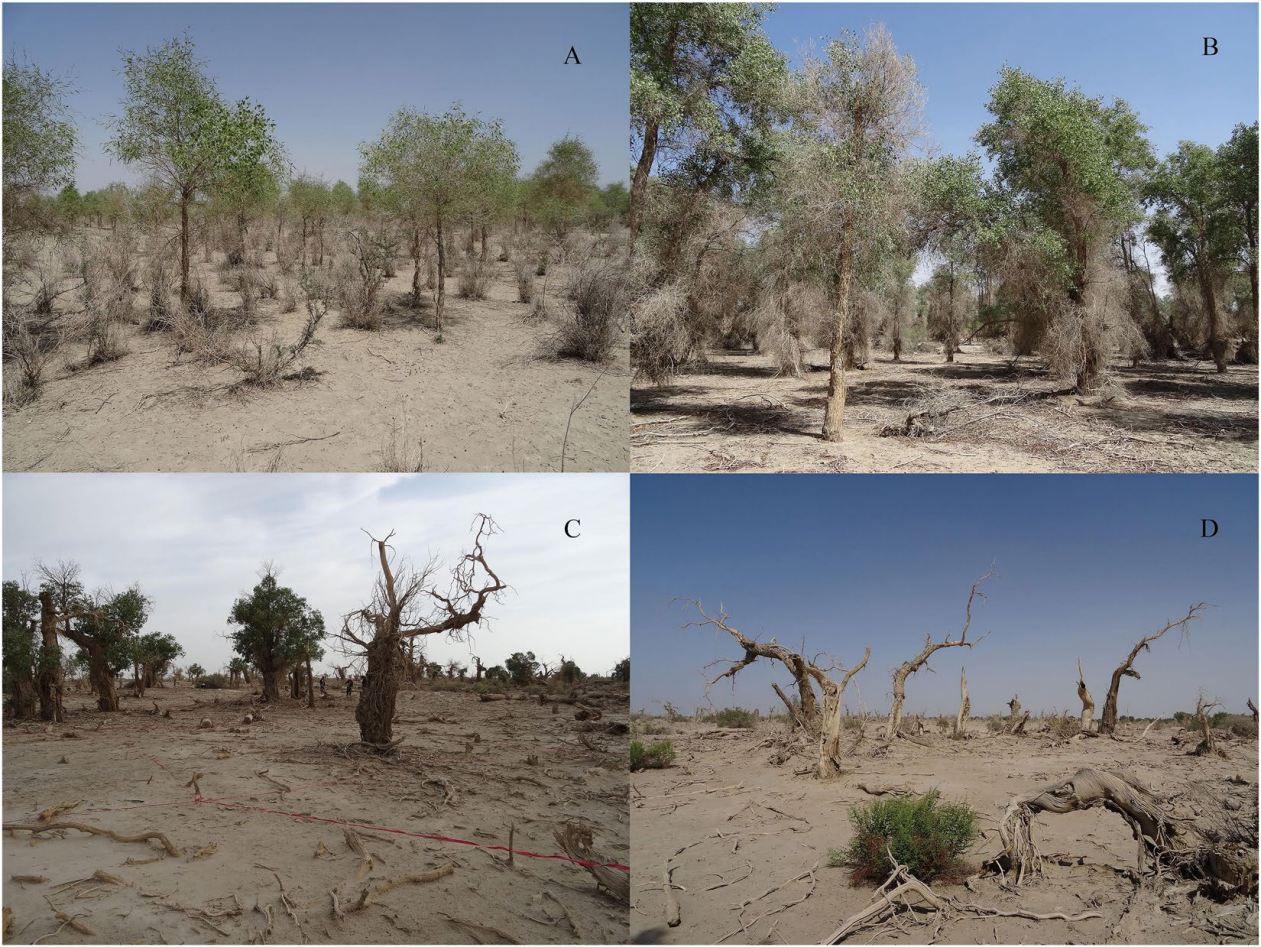
*P. euphratica* forest photos showing the different growth and death stages: (**A**) Juvenile forest plot, (**B**) Mature forest plot, (**C**) Dying forest plot, and (**D**) Dead forest plot.

*P. euphratica* is typically the dominant tree species in this area, and, in our four plots, it was the only tree species (Fig. 1). The main shrub species in our plots were *Tamarix ramosissima* Ledeb., *T. chinensis* Lour., *Lycium ruthenicum* Murr., and *Halimodendron halodendron* (Pall.) Voss. The main herb species were *Alhagi sparsifolia* Shap., *Suaeda rigida* Kung et G. L. Chu, *Glycyrrhiza uralensis* Fisch., *Asparagus persicus* Baker, *Oxytropis glabra* (Lam.) DC.*, Kalidium foliatum* (Pall.) Moq., and *Karelinia caspia* (Pall.) Less.. There were very few *T. ramosissima* individuals in the juvenile and mature plots; however, in the dying forest and dead forest plots (Fig. 1C,D), *T. ramosissima* was notably more common and generally in good form, suggesting that the *T. ramosissima* species would replace the dying *P. euphratica*.

### Size-class structure and growth status

**Figure 2:**
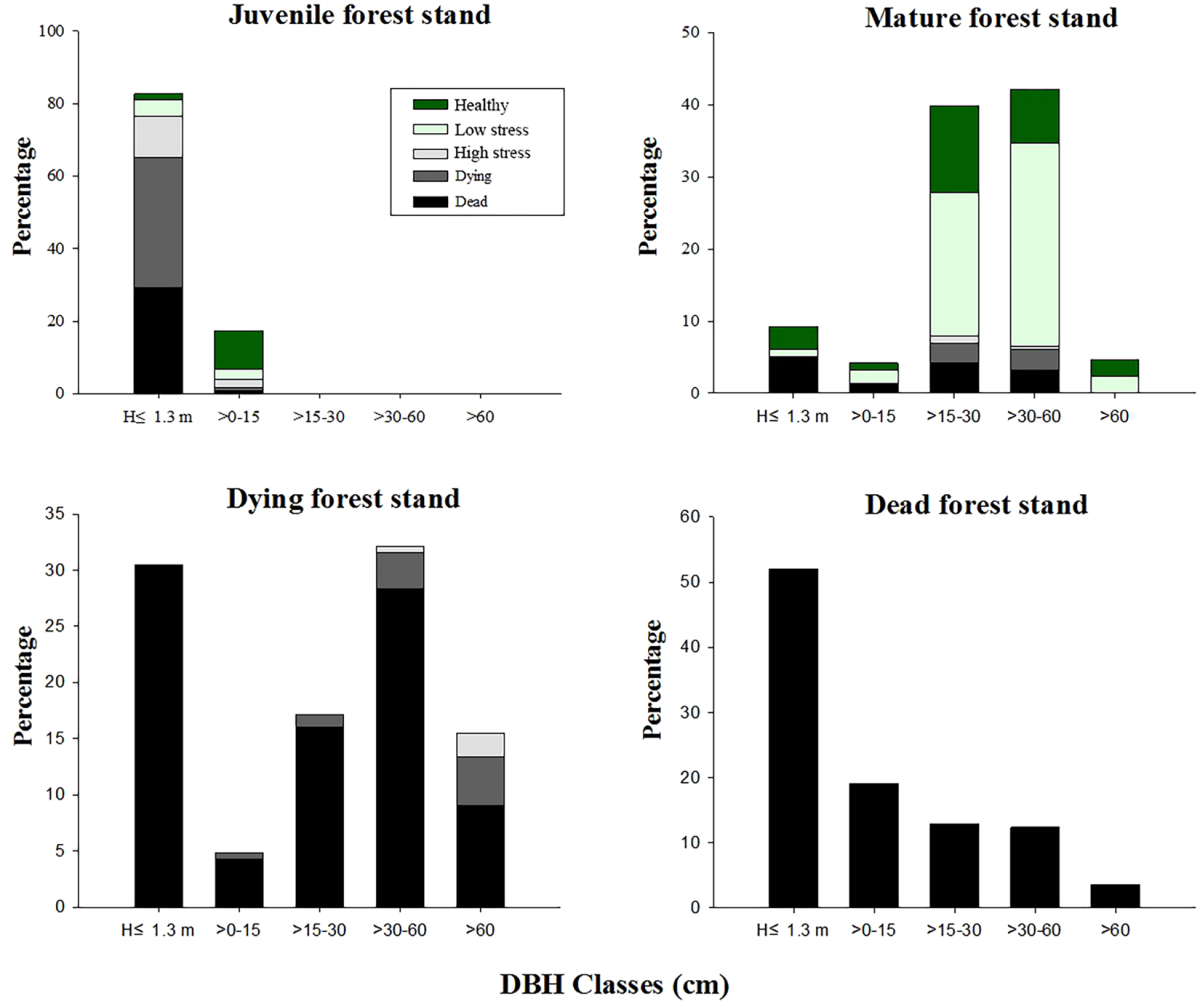
Growth status and size-classes of *P. euphratica* in the different plots. H ≤ 1.3 m indicates trees that were equal to or less than 1.3 m in height. (**A**) Juvenile forest plot, (**B**) Mature forest plot, (**C**) Dying forest plot, and (**D**) Dead forest plot.

In the juvenile forest plot (Fig. 2A), 0.41 km from the river, the majority (82.7%) of the trees (live and dead) were saplings equal to or shorter than 1.3 m. Over one third (35.4%) of these saplings had already died, and 43.5% of these saplings were dying. Among the larger trees (height > 1.3 m), only 9.2% were dead or dying.

In the mature forest plot (Fig. 2B), 1.51 km from the river, 9.3% of the trees were saplings equal to or shorter than 1.3 m, while 55.0% of these small saplings had already died. In contrast, among the larger trees (height > 1.3 m), 8.8% were dead or dying, while 22.7% were evaluated as healthy trees. Nevertheless, on this mature forest plot, just over one kilometre farther from the river than the juvenile plot, 60.2% of the trees showed clear signs of drought stress (low stress, high stress, and dying).

**Figure 3:**
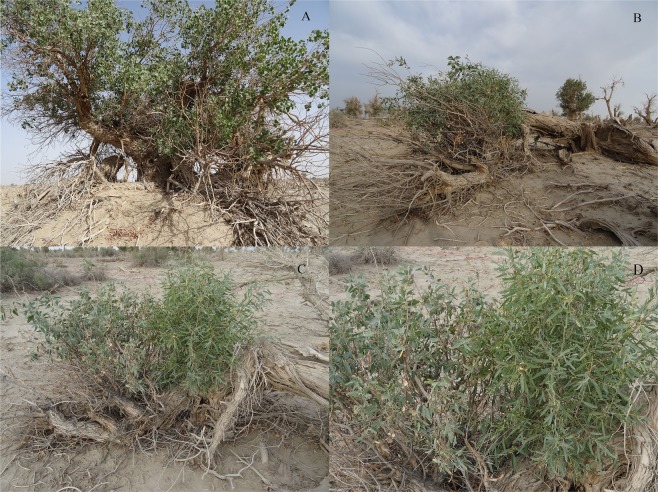
Shape-shifting of *P. euphratica* trees in the dying forest plot: (**A**) Tree with reduced crown and expanding lower branches, (**B**) Fallen trunk with clump of root suckers, (**C**) Fallen trunk with remainder clump of small branches and a new clump of root suckers, and (**D**) Close-up of small branch clump and root sucker clump to show differences in leaf shape and colour between the two clumps.

In the dying forest plot (Fig. 2C), 88.2% were dead trees with the remaining live trees being either high stress or dying. It is abundantly clear that the severity of the drought stress in this forest plot is beyond the level at which the *P. euphratica* trees can survive with their typical height and shape. Many of the high stress and dying trees showed strong reduction of their crowns and subsequently the main trunks of many fell down. Some dying trees with reduced crowns showed expansion of lower branches (Fig. 3A), and others, even those whose trunks fell over, showed a proliferation of sucker shoots (Fig. 3B,C). The sucker shoots of *P. euphratica* typically showed a change in leaf shape, texture, and colour (Fig. 3D shows the shape and colour of the leaves on the sucker shoots on the right compared to the normal leaves on the remaining small tree branches on the left).

**Figure 4:**
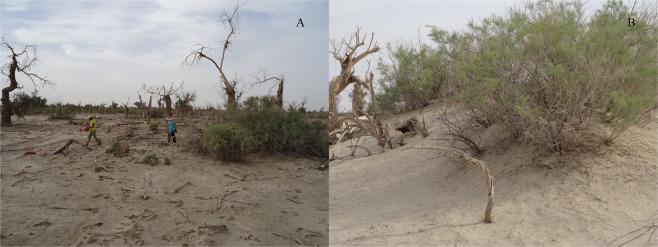
*T. ramosissima* clumps in the Dying (**A**) and Dead (**B**) forest plots.

In the dead forest plot (Fig. 2D), while dead trees of all size-classes were found in this plot, about half of the dead trees (52.1%) were shorter than 1.3 m, which most likely represented failed root suckers (examples can be seen in Fig. 4B).

In contrast to the many dying and dead *P. euphratica* trees in the dying and dead forest plots, a number of *T. ramosissima* shrub clumps were scattered over these plots with most of them appearing green without showing many dying leaves (Fig. 4A,B). Under high drought stress conditions in the dying and dead forest plots, the *T. ramosissima* shrubs were clearly surviving better than the *P. euphratica* trees.

### Spatial distribution patterns of live and dead trees

**Figure 5:**
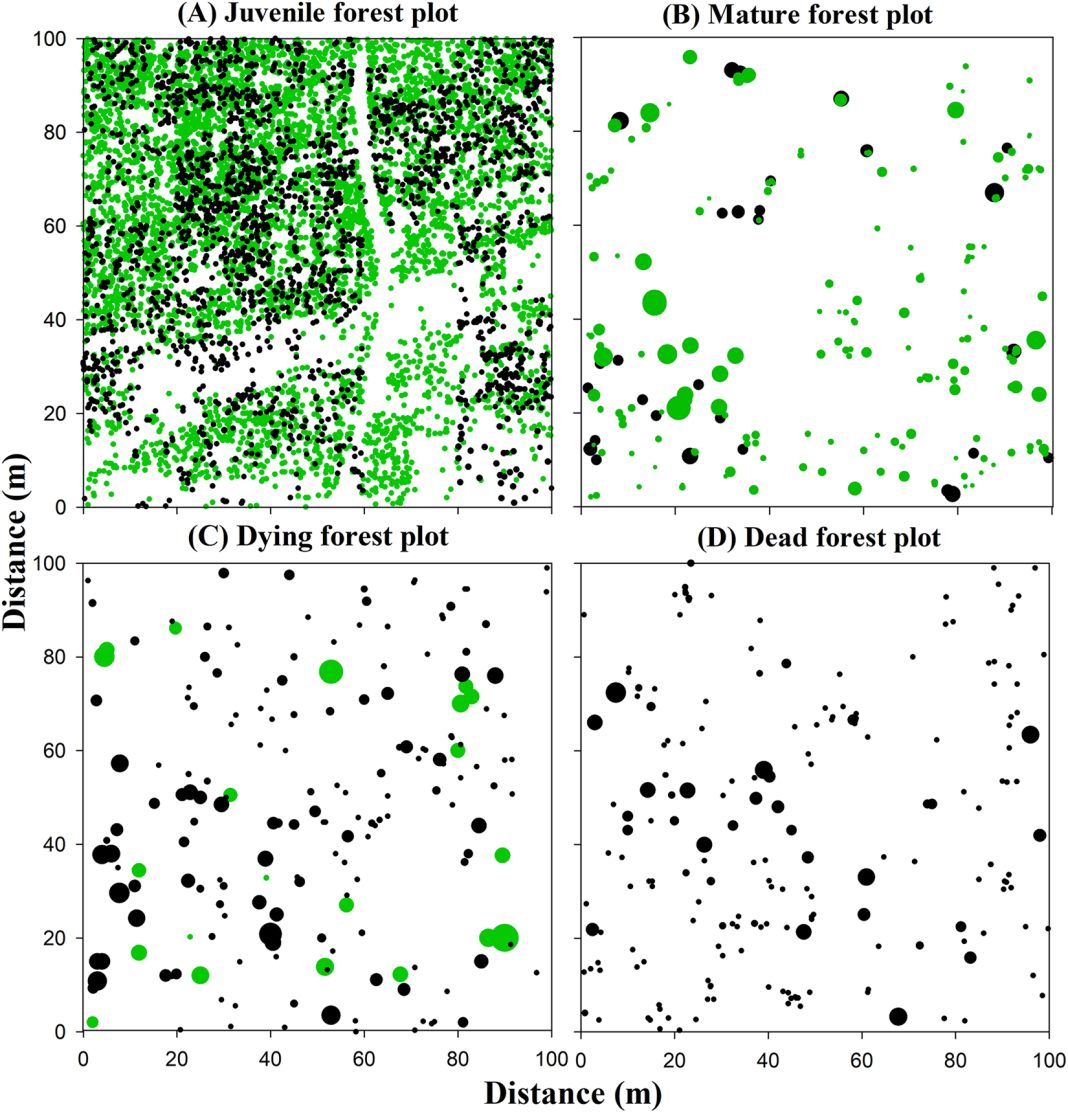
Map of four 1-ha forest plots showing the location and size of all *P. euphratica* trees: Green colour represents live trees and black colour represents dead trees. The size of trees is indicated by symbol sizes proportional to the DBH or basal diameter. Smallest sizes in each of the plots represent trees equal to or less than 1.3 m in height. (**A**) Juvenile forest plot, (**B**) Mature forest plot, (**C**) Dying forest plot, and (**D**) Dead forest plot.

Locations and sizes of *P. euphratica* in the different stages are shown in Fig. 5. From the juvenile forest plot (Fig. 5A) to the mature forest plot (Fig. 5B) and dying forest plot (Fig. 5C), densities of live trees became dramatically lower. From the mature forest plot (Fig. 5B) to the dying forest plot (Fig. 5C) and the dead forest plot (Fig. 5D), more dead trees were present.

**Figure 6:**
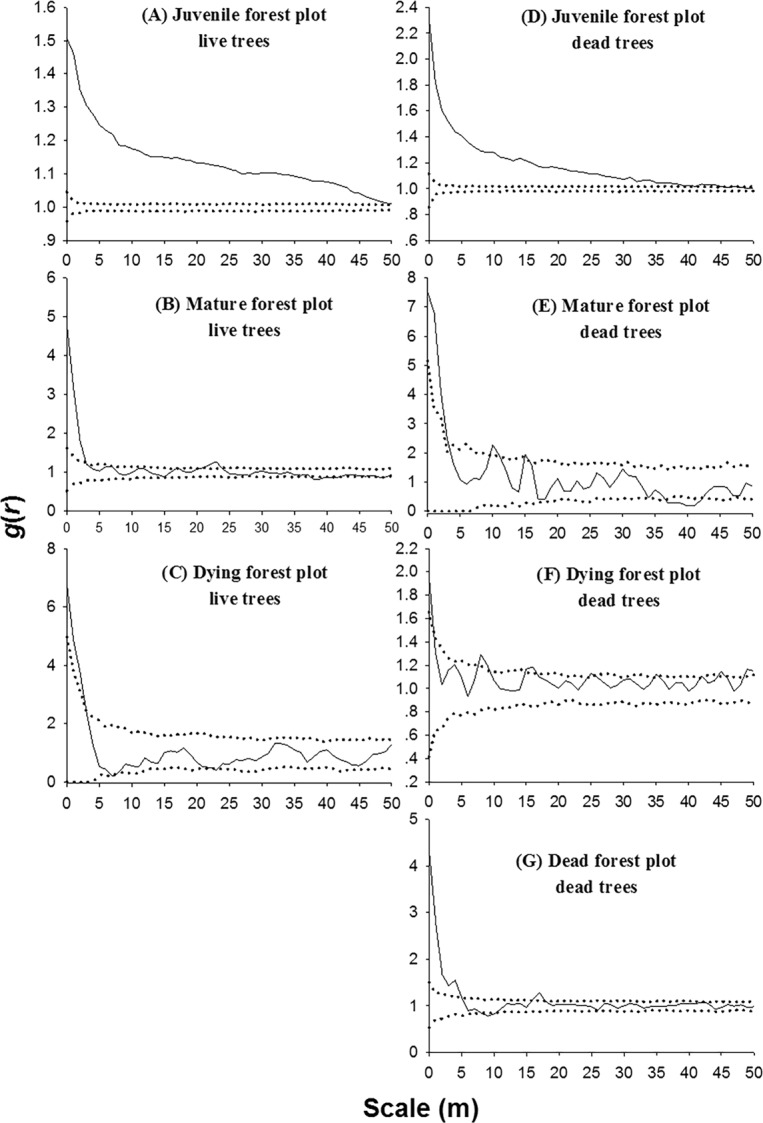
Spatial distribution patterns of the live trees and the dead trees in the different stages. Solid black lines indicate the *g*_12_(*r*); thin dotted lines indicate the upper and lower limits of the 95% simulation envelope under the CSR null model. Points above the 95% envelope indicate aggregated patterns, points within the 95% envelope indicate random patterns, and points below the 95% envelope indicate regular patterns.

In the juvenile forest plot, live trees were significantly aggregated at all scales (*p* < 0.05) (Fig. 6A); while, in the mature forest and dying forest plots, live trees were significantly aggregated at scales of 1–4 m and 1–3 m, respectively, and mainly randomly distributed at greater scales (*p* < 0.05) (Fig. 6B,C). In the juvenile forest plot, dead trees were significantly aggregated at scales < 45 m (*p* < 0.05) (Fig. 6D); while, in the mature forest, dying forest, and dead forest plots, dead trees showed aggregated patterns only at small scales of 0–4 m, 0–2 m, and 0–5 m, respectively, and mainly showed random patterns at scales larger than those (*p* < 0.05) (Fig. 6E–G).

### Spatial associations of live and dead trees

**Figure 7:**
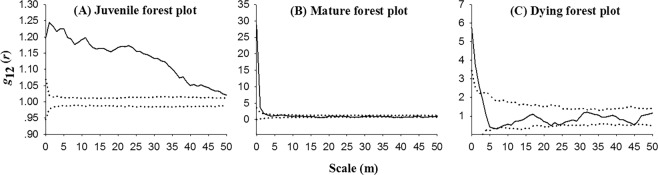
The *g*_12_(*r*) values of intra-specific spatial associations between live trees and dead trees in the different stages. Solid black lines indicate the *g*_12_(*r*); thin dotted lines indicate the upper and lower limits of the 95% simulation envelope under the pattern 1 and pattern 2 CSR null model.

In the juvenile forest plot, live trees and dead trees showed a significantly positive association at all scales (*p* < 0.05) (Fig. 7A), indicating a density-dependent effect in the plot, i.e. tree interactions are likely to respond directly to their immediate neighbouring trees, thus tree death is mainly caused by tree density effect^31–33^. While in the mature forest and dying forest plots, live trees and dead trees showed a significantly positive association only at small scales of 0–3 m (*p* < 0.05) and showed independence at larger scales (Fig. 7B,C), indicating that the density-dependent effect between live trees and dead trees largely only occurred at small scales.

### Random mortality hypothesis

**Figure 8:**
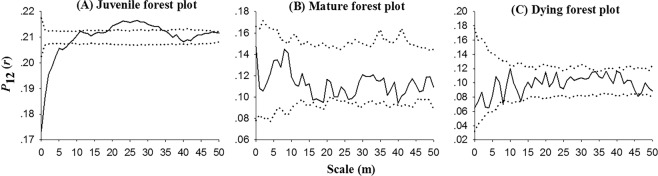
Probability of survival of trees depending on distances from dead trees to live trees in the different stages. We compared the spatial pattern of dead trees (index 1) and live trees (index 2) to the model of random mortality using a test statistic that gives the probability of survival *P*_*a*,2_(*r*) of trees at distance *r* from live trees. Solid black lines indicate the *P*_12_(*r*); thin dotted lines indicate the upper and lower limits of the 95% simulation envelope under the random labelling null model.

In the juvenile forest plot, mortality was significantly non-random at scales of 0–7 m (*p* < 0.05), i.e., the probability of tree survival increased rapidly from the scales of 0–7 m as the relative densities of trees decreased at increasing distances (Fig. 8A), indicating that tree death was significantly related to density-dependent effect at closer distances of trees in the juvenile forest stage. In the mature and dying forest plots, mortality was spatially random at all scales (Fig. 8B,C), i.e., dead trees did not change the spatial distribution patterns of live trees, indicating that tree death was in effect a random event and generally unrelated to density-dependent effects in the mature forest and dying forest stages.

### Correlates of tree survival

Comparing the juvenile plot with the mature plot and with the combined dying and dead plots, the height of trees showed a significantly positive correlation with tree survival (*p* < 0.001), and greater distances from the river showed significantly negative correlations with tree survival (*p* < 0.01) (Table 1).

**Table 1:** Effects of tree height and distance from the Tarim River on tree survival over the different plots^a^.

Factors	Estimate	Std. Error	*z* value	*P*_*r*_ (>|*z*|)	
Height	0.9743	0.1371	7.107	1.19e-12	***
Distance from the Tarim River	−15.3951	−7.0035	−2.198	0.0279	*

^a^Juvenile plot + mature plot + (dying plot + dead plot).

Significant codes: *p < 0.05; ***p < 0.001.

When we examined these survival rates within each of the three plots with live trees (Table 2), DBH and height had significantly positive correlations with tree survival (*p* < 0.05) in the juvenile forest and mature forest plots, indicating that the trees that were larger or taller had significantly higher survival rates than smaller and shorter trees in the juvenile and mature forest plots. In the dying forest plot, the GLMM analysis showed no significant correlation between tree survival and DBH or tree height (Table 2).

**Table 2:** Effects of DBH and tree height on tree survival in the different stages using GLMM analysis.

	Juvenile forest plot	Mature forest plot	Dying forest plot
DBH	0.033*	0.002**	0.667 NS
Height	1.27e-15***	1.3e-07***	0.287 NS

Significant codes: *0.01 ≤ *p* < 0.05; **0.001 ≤ *p* < 0.01; ***p < 0.001; NS, not significant.

We believe the lack of a significant correlation between tree survival and DBH and height in the dying forest plot was due to a limitation of the GLMM analysis to deal with the small sample size of the live trees in the dying forest plot. If the actual live and dead trees of the dying forest shown in Fig. 2 are examined, it is evident that, while the dead trees showed nearly equal numbers of small and large trees, the surviving live trees were almost entirely larger in DBH and height. Comparing the 22 live trees to the dead trees, we found that 19 of the 22 live trees had DBHs greater than the median DBH size of the dead trees (*p* = 0.001, binomial test) and that all 22 of the live trees had heights greater than the median height of the dead trees (*p* < 0.0001, binomial test). Thus, the results of the binomial tests confirmed that the survival of the 22 remaining live trees was significantly correlated with their greater sizes as shown by their DBHs and heights.

## Discussion

### Tree death in the different stages

In the juvenile forest plot, tree death was not random but rather significantly related to density-dependent effects. Among dead trees, 97.5% were equal to or less than 1.3 m, indicating that trees up to 1.3 m high could be in a critical phase of survival. The high intensities of aggregation of juvenile trees, especially at smaller scales, would be expected to experience high density-dependent mortality. This expectation was supported by the fact that juvenile dead trees showed much higher intensities of aggregation at small scales compared to juvenile live trees (compare Figs. 6D to 6A). Thus, the higher mortality or lower survival probability of juvenile trees at smaller scales led to a reduction of the density of the remaining juvenile live trees. This spatial pattern of higher mortality at the smaller scales is consistent with thinning due to the density-dependent effect in the juvenile forest plot^18,34^.

However, in the other stages, tree mortality was generally random and not related to density-dependent effects. We found that the higher death rates of trees were significantly related to sites at greater distances from the Tarim River (*p* < 0.01). This relationship could be explained by the fact that groundwater levels generally decrease at greater distances from the Tarim River^12,35^. Mortality in mature, dying, and dead forests was likely to have been caused primarily by drought. With increasing distances from the Tarim River, the *P. euphratica* forest types changed from juvenile forest (0.41 km from the river) to mature forest (1.51 km from the river) to dying and dead forest (> 5 km from the river). Those series of changes illustrate dramatic differences in survival of *P. euphratica* over the increasingly harsh dry environments at greater distances from the river^10^. With the increasing distances from the Tarim River, the distributions of *P. euphratica* forests were reduced, *P. euphratica* growth decreased, and dying and dead forests became the principal type of *P. euphratica* forests in the Tarim River basin^21^.

In the dying and dead forest plots, all trees equal to or shorter than 1.3 m were dead. This result is consistent with another study where under the influence of drought stress with heavy sand dunes, there were many dead juvenile trees in *P. euphratica* forest^36^. We assume that, when facing extreme groundwater scarcity, *P. euphratica* forests will produce more new individuals as root suckers (asexual reproduction) rather than attempting to produce seedlings.

### Shape-shifting of *P. euphratica* under extreme drought stress

Under high drought stress conditions where *P. euphratica* was dying or had died (Fig. 1C,D), clumps of the shrub *T. ramosissima* were shown to have been replacing *P. euphratica* (Fig. 4A,B). Compared with *P. euphratica*, the *Tamarix* clumps have several adaptations that permit them to continue to survive at high levels of drought stress: (1) these shrubs form relatively low dense shrubby clumps to reduce exposure to breezes and wind that could increase evaporation from the leaves^37^; (2) they have narrow, leathery-textured fuzzy leaves that reduce rates of evaporation and capture more nightly dew^37^; (3) they have deep tap roots that can potentially reach groundwater at greater depths^38^; and (4) they are more resistant to the effects of soil salinization^3,39^.

While the *P. euphratica* do also have deep tap roots like *Tamarix*, other features of *P. euphratica*, such as their higher canopies above thick trunks and wider, thinner leaves, become disadvantageous under severe drought stress^38^. When these poplars faced extreme drought (as shown in Fig. 1C,D), we observed two major changes leading these poplar trees to take on the apparent shape of shrubs: (1) their canopies begin to die back, which in some cases included an expansion of lower branches (Fig. 3A), and (2) they begin to foster multiple root sprouts (see Fig. 3B,C). This result is consistent with the eco-morphology responses of *P. euphratica* under extreme drought stress^2,35^. It may well be that when there is too little water and too much evaporation, these poplars may use whatever deep soil water or groundwater that their roots can reach to foster the production of clumps of root sprouts as well as altering the shape and texture of the leaves of the root sprouts to reduce evaporation and increase the ability to capture nightly dew^40^ (see Fig. 3D). In the face of this extreme drought, poplar trees appear to be doing this kind of shape-shifting into a more shrub-like form, with the clumps of root sprouts replacing the high canopies and drawing on the existing deep and widely spread root systems of the older trees^35^ (see the two examples in Fig. 3B,C).

While the shape-shifting changes from the greatly stressed poplar trees to shrub-forms did not appear to have halted the widespread dying of these trees in our dying forest plot (Fig. 2C), we suggest that the shift to shrub-forms may serve to buy more time (a “sit-and-wait” strategy) for these poplars to wait for any possible return of any source of water, whether through rain or flooding or a rise in the groundwater. While the situation we encountered in our dying forest plot documented the poplars near the end of struggling against severe drought stress, other studies have noted *P. euphratica* commonly producing root sprouts under levels of drought stress where seeds and saplings would not be able to survive^35,36,41^. This suggests that the shape changes associated with production of clumps of root sprouts is an evolutionary response that may sometimes be adaptive under severe but unpredictable drought stress. It is worth noting that in arid desert lands and scrublands in Australia a number of tree species often grow multiple trunks in the form of shrubs, such as mallees of certain eucalypt species like *Eucalyptus socialis* (Myrtaceae)^42^, several species of *Banksia* like *B. rosserae* (Proteaceae)^43^, and *mulgas* like *Acacia aneura* (Fabaceae)^44^.

### Two basic assumptions of the study related to water and their limitations

Our study included two basic assumptions relevant to water availability. For assumption 1, we assumed that forest plots at earlier stages of development (i.e., sites with healthy growing trees) have higher groundwater levels than forest sites showing strong signs of drought distress. Thus, we assumed that our juvenile forest plot with a high density of small saplings likely had a higher groundwater level than the mature forest plot that included a number of healthy trees but very few live small trees. Likewise, we assumed that the mature forest plot likely had a much higher groundwater level than the dying and dead plots which showed strong signs of death from severe drought stress.

For assumption 2, we assumed that forest sites at greater distances from the river very likely had lower groundwater levels. This assumption included two caveats: 2 A) we assumed that while we expected groundwater levels to decrease at greater distances from the Tarim River, ground water levels likely showed some degree of variation within relatively short distances at any given site; and 2B) we assumed that groundwater levels would show higher groundwater levels than expected at sites near cut-offs.

While the juvenile and mature forest sites were within 1.5 km of the Tarim River, the dying and dead plots were both at sites greater than 5.6 km from the Tarim River. And, it was significant that the dying forest plot was actually about 0.2 km further from the Tarim River than the dead forest plot. While these two forest plots were only 0.37 km apart and both sites showed signs of extreme drought distress, the dying forest site did not appear as severely affected by drought stress compared to the dead forest site as one can see by examining the photos of each site in Fig. 1C,D. We found no signs or evidence of any cut-offs anywhere near these two sites nor any signs of forestry activity. Therefore, we suggest that the obvious differences between the two sites were likely due to local small-scale differences in their groundwater levels (Assumption 2 A), as well as the possibility of some other small-scale undetected differences between those two sites, even though they were only 0.37 km apart. If studies like this were to include examination of factors such as groundwater levels, soil water, and soil salinity, they would expand our understanding of the forest pattern and development in this dynamic and challenging desert region.

### Implications

Since we found different spatial patterns and structural properties in the different stages, management for the *P. euphratica* forests should be different. Thinning can be incorporated in the juvenile stage of *P. euphratica* forests management, since tree death is likely mainly caused by density-dependent effects in the juvenile forest stage. For mature and older stages of *P. euphratica* forests, groundwater control or water supply is very critical, since in those stages tree death is independent of density effects but strongly related to water availability. We found that *P. euphratica* forests would sprout root suckers under extreme drought. In the face of extreme drought, poplar trees appeared to be doing a kind of shape-shifting into a more shrub-like form, with clumps of root sprouts with narrow, leathery-textured fuzzy leaves replacing the high canopies and drawing on the existing deep root systems of the older trees. Under extreme drought stress, the shift to more shrub-like forms may extend their time to wait for any favourable change. The dying forests showing these shrub-like forms with clumps of root sprouts may represent the last favourable chance where an appropriate supply of water may serve to save the dying forests. We contend that the dynamics and ecological effects of dying and dead *P. euphratica* forests should be the focus of more research to assess their ecological values.

## Methods

### Study sites

**Figure 9:**
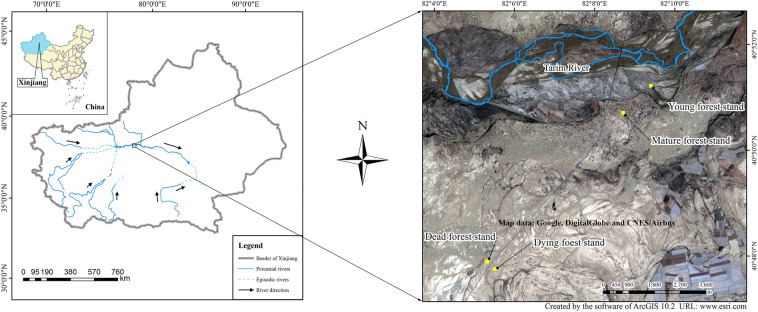
Study sites and the distribution of the four plots. The image is from Google Earth (© Google, DigitalGlobe and CNES/Airbus).

Our study sites were located along the upper reaches of the Tarim River at the northern margin of the Tarim Basin of the Xinjiang Uygur Autonomous Region, China (Fig. 9). This area is dominated by a typical continental temperate arid climate characterized by hot summers and cold winters with a mean annual temperature of 10.8 °C. The Tarim Basin is one of the most arid areas in China with a mean annual precipitation of 40 mm and a mean annual evaporation of 2,590 mm^45^.

### Data collection

In July 2015, we selected four typical *P. euphratica* forest plots (100 m × 100 m) representing four different growth and death stages: juvenile forest (mean height of trees is lower than 2 m), mature forest (mean height of trees is higher than 9 m), dying forest (majority of trees are dead), and dead forest (all trees are dead) (Figs. 1 and 9). For all *P. euphratica* trees in each plot, we measured height, calculated coordinates, and assessed relative tree status. To access the relative tree status in each of the plots, we classified tree vitality into five categories based on losses among crown and branches: we classified tree vitality into five categories based on losses among crown and branches: healthy (intact tree form), low stress (less than 10% loss), high stress (10% to two-thirds loss), dying (more than two-thirds loss), and dead (standing dead trees and fallen logs).

For trees taller than 1.3 m, we measured DBH at 1.3 m height. For stumps (standing dead trees shorter than 1.3 m), we measured their basal diameter at 0.1 m height the ground. For fallen logs, we also measured their DBHs and locations from the trunk base.

For the plots, the point (*x*, *y*) = (0, 0) was located in the bottom left corner. The location of each individual within the plots was mapped on the *xy*-plane; the coordinates for a given tree locate the centre of the main stem to the nearest 0.1 m. Tree height was determined by a measuring stick if the tree was ≤ 2 m and with a clinometer if the tree was > 2 m. Characteristics of the plots are shown in Table 3.

**Table 3:** Basic information of the four *P. euphratica* forest plots.

Forest plot type	Elevation (m)	Distance from the Tarim River (km)	Density of live trees (stem/ha)	Density of dead trees (stem/ha)	Tree height (m)
Juvenile forest plot	975	0.41	5,316	2,277	1.1 ± 0.8
Mature forest plot	979	1.51	186	30	9.7 ± 4.23
Dying forest plot	984	5.86	22	165	2.4 ± 2.2
Dead forest plot	984	5.67	0	194	1.6 ± 1.5

### Spatial distribution pattern analysis

Different ecological processes may leave a particular signature on the spatial arrangement of individuals; therefore, studying spatial patterns may help to reveal the importance of underlying mechanisms^30^. We used the pair correlation function *g*(*r*)^46^ as a summary statistic to describe the spatial correlation structure of the univariate point patterns observed at our study site. Since the plots were generally flat (Fig. 2), we assumed environments were homogeneous and chose homogeneous null models^29^ for our point-pattern analysis.

We assessed the spatial distribution patterns of *P. euphratica* trees (live and dead) in the four different growth and death patterns represented by the forest stages in the four plots shown in Fig. 5. In order to make such an assessment, we used the pair correlation function *g*(*r*) as the test statistic and implemented a null model based on a homogeneous Poisson process, hence the intensity, *λ*, varies with the location (*x*, *y*).

### Spatial association analysis

To investigate the relationship between the live and the dead trees in the different stages, we used the independence null model^47^, which tests for independence between two patterns. If the observed bivariate pair-correlation function *g*_12_(*r*) was above, below, or between the simulation envelopes, the patterns showed positive association, negative association, or independence, respectively.

### Test of the random mortality hypothesis

Due to year-round arid conditions where these forests exist, decomposition processes are very slow such that many of dead trees of *P. euphratica* can remain standing for several decades^1^. Therefore, we hypothesize that the spatial patterns of *P. euphratica* forests do not change dramatically after trees die. We used the test statistic *P*_12_(*r*)^48–50^ to test random mortality of trees in the different stages and to explore probability of survival of trees depending on distances from dead trees to live trees. To explore possible non-random spatial structures in tree mortality, we used random labelling as a null model^51–53^. The framework of ‘marked point patterns’ is required to identify the spatial structures in the process that assigned a label ‘mortality’ to the trees^53^. This null model assumes that mortality acted as a random process over a given tree pattern, i.e., the *n*_2_ dead trees of a stand are assumed to be a random subset of the joined pattern of the *n*_2_ dead and *n*_1_ live trees (1 referring to live and 2 to dead trees)^31,48,51,54^. The test of the random mortality hypothesis was conducted by using a Monte Carlo implementation of random labelling from the observed data, then randomly re-sampling sets of *n*_2_ trees from the joined pattern of dead and live trees to generate simulation envelopes of the test statistic. The software Programita^28^ provided the appropriate test statistics of *P*_12_(*r*), which was also independently proposed by de la Cruz *et al*.^55^.

All the spatial pattern analyses above were done using the software *Programita*^47,49^. Statistical significance of the functions above was determined with the Monte Carlo simulations^56^. To assess departures from the null model, we compared the *g* functions spatial patterns created by 199 simulations of the heterogeneous Poisson null model with an upper and lower simulation envelope encompassing approximately 95% of the simulation values with the highest five simulation values below the upper simulation envelope and the five lowest simulation values above the lower simulation envelope^49^. Note, that the simulation envelopes cannot be interpreted as confidence intervals for formal hypothesis testing because type I error inflation may occur due to simultaneous inference (i.e., tests at many spatial scales: the distances from focal trees to other trees)^56^.

### Generalized linear mixed-effect model analysis

We modelled individual tree survival as a function of tree size (DBH) and height and the distance of the plots from the Tarim River using logistic generalized linear mixed-effects model (GLMM)^57,58^. Individual survival was a binary variable (i.e., live or dead, coded as 1 or 0, respectively). The GLMM was constructed using the lme4 package^59^ in R software (V. 3.3.1)^60^ to model the probability of tree survival as a function of explanatory variables with binomial errors.

We combined the dying forest and dead forest plots together to assess the effects of distance from the river on tree survival, since these two plots 1) were very similar in their dying/death stages, 2) had low densities of trees (with only 22 live trees in the dying forest plot), 3) were spatially in close proximity to each other, and 4) were both nearly the same distance from the river and with both being > 4 km from the mature and juvenile forest plots.

Using a GLMM, we compared tree survival and death from the juvenile forest plot to the mature forest plot and the combined dying and dead forest plots. We tested for correlations between tree survival and death and 1) distances of the forest plots from the Tarim River and 2) tree size using tree height. We could not test for a correlation across all the forest plots using tree DBH, since only 17.3% of juvenile trees were taller than 1.3 m in the juvenile forest plot. In addition, we did GLMM analysis in the juvenile, mature, and dying forest plots separately to infer the effects of tree sizes (DBH and height) on tree survival in the different stages.

In the dying forest plot, there were 165 dead trees and only 22 live trees. Since the number of live trees was relatively quite small, it was difficult to validate relative differences between the live trees and more numerous dead trees. Therefore, we chose to use a special nonparametric test, the binomial test, to determine if the live trees were significantly larger than the median of the dead trees in the dying forest plot.
